# Prehospital and intra-hospital time delays in posterior circulation stroke: results from the Austrian Stroke Unit Registry

**DOI:** 10.1007/s00415-016-8330-x

**Published:** 2016-11-07

**Authors:** Peter Sommer, Leonhard Seyfang, Alexandra Posekany, Julia Ferrari, Wilfried Lang, Elisabeth Fertl, Wolfgang Serles, Thomas Töll, Stefan Kiechl, Stefan Greisenegger

**Affiliations:** 1 Department of Neurology, Krankenanstalt Rudolfstiftung, Vienna, Austria; 2 Danube University Krems and Gesundheit Österreich GmbH/BIQG, Vienna, Austria; 3 Department of Neurology, Krankenhaus Barmherzige Brüder, Vienna, Austria; 4 Department of Neurology, Medical University of Vienna, Waehringer Guertel 18-20, 1090 Vienna, Austria; 5 Department of Neurology, Medical University of Innsbruck, Innsbruck, Austria

**Keywords:** All cerebrovascular disease/stroke, Infarction, Cohort studies, Intravenous thrombolysis, Posterior circulation stroke

## Abstract

Therapeutic effect of recombinant tissue-plasminogen activator (rt-PA) is time dependent. There is limited evidence whether localization of stroke within the posterior circulation (PCS) is associated with a treatment delay. We aimed to analyze within a nationwide multicenter cohort whether duration of pre- and intra-hospital patient management differs between patients with PCS and anterior circulation strokes (ACS). We studied onset-to-door-times (ODT) and door-to-needle-times (DNT) of all patients with acute ischemic stroke (IS) enrolled in the Austrian Stroke Unit Registry according to infarct localization. Classification into PCS and ACS was based on clinical presentation applying the criteria used in the Oxfordshire Community Stroke Project. Relationships between ODT, respectively, DNT and explanatory variables were modeled by multivariate linear regression. Between 2003 and 2015, 71010 patients with IS were enrolled, 11,924 with PCS and 59,086 with ACS. Overall, the ODT was significantly longer in PCS: median (IQR): 170 (25th, 75th‰: 79,420) min versus 110 (60,240); *p* < 0.001; this finding held true in multivariable analysis. In 10535 rt-PA-treated patients (1022 PCS/9832 ACS), ODT and DNT were significantly longer among those with PCS: ODT: median: 80 min (55,120) versus 72 (50,110), *p* < 0.001; DNT: 57 (35.90) versus 45 (30.67), *p* < 0.001. In the multivariate model, PCS was significantly associated with delay in the DNT. In conclusion, in this large nationwide cohort, patient management was significantly slower in PCS as compared to ACS. Increasing awareness about these delays and further elaboration of the underlying causes may translate into higher proportions of patients with PCS receiving rt-PA.

**Electronic supplementary material:**

The online version of this article (doi:10.1007/s00415-016-8330-x) contains supplementary material, which is available to authorized users.

## Introduction

Approximately one-fifth of ischemic strokes occur in the posterior circulation [1]. However, compared to anterior circulation strokes (ACS) percentage of patients with posterior circulation strokes (PCS) receiving intravenous recombinant tissue-plasminogen activator (rt-PA) was lower across different stroke registries even though approval does not exclude specific localizations such as posterior circulation [2–5]. This difference might be influenced by underrepresentation of PCS in randomized controlled trials [6–10]. In addition, clinical symptoms of PCS differ markedly from ACS and may be misinterpreted in clinical practice [1, 11]. This may account for delays in the prehospital setting but also after arrival in hospital.

While only few smaller studies reported time intervals before thrombolysis (i.e., onset-to-treatment-times, OTT) stratified by ACS and PCS [3, 5, 12] only one evaluated onset-to-door-(ODT) and door-to-needle (DNT)-times specifically [12]. As the therapeutic effect of rt-PA treatment in stroke is highly time dependent, detailed knowledge of pre- and intra-hospital time intervals and factors associated with delays is crucial to adapt treatment algorithms [13–15]. Uncertainties in relation with transient symptoms related to the posterior circulation can result in lower rates of diagnosis of TIAs [16]. Therefore, both pre- and intra-hospital delays might arise. Misdiagnosis and delays may have significant implications for treatment and could have potential medicolegal consequences.

We, therefore, aimed to analyze in a large nationwide cohort of patients with acute ischemic stroke (Austrian Stroke Unit Registry, ASUR) whether pre- and intra-hospital time intervals differ between patients with PCS and ACS.

## Methods

### Patients

The methodology of the ASUR including definitions of clinical variables has been described previously [17, 18]. Briefly, the ASUR is a network including all Austrian stroke units collecting data on standard characteristics and acute management of all patients with stroke admitted to one of currently 36 centers since 2003. Data acquisition and rating of clinical scales are performed by experienced stroke neurologists. Clinical evaluations are conducted at the time of admission to and at discharge from the stroke units. The resulting web-based database includes online plausibility checks. Symptomatic intracerebral hemorrhage (SICH) was rated according to National Institute of Neurological Disorders and Stroke criteria (any computer tomography (CT) or magnetic resonance imaging (MRI) documented bleeding with clinical deterioration of at least one point on the National Institute of Health Stroke Scale (NIHSS) or leading to death within 7 days) [6]. The initial stroke syndrome was categorized according to the Oxfordshire Community Stroke Project (OCSP) [19].

The ASUR is part of a governmental quality assessment program for stroke care in Austria financed by the Federal Ministry of Health. It is based on the federal law promoting quality in health (Gesundheitsqualitätsgesetz). All data are anonymized and centrally administered by the Gesundheit Österreich GmbH—the national research and planning institute for health care and a competence and funding center of health promotion. All scientific analyses are approved and supervised by an academic review board [17, 18, 20].

### Statistical methods

We studied ODT and DNT of enrolled patients with ACS and PCS. Kruskal–Wallis one-way analysis of variance was performed for univariate analysis of time intervals stratified by infarct localization. Associations of ODT and DNT with other clinical variables were analyzed by multivariate linear regression models. The model specification was done using bidirectional stepwise variable selection procedure, optimizing the Bayesian Information Criterion. Following potentially explanatory variables were included into the selection procedure for the ODT model: infarct localization (ACS or PCS), type of transport (ambulance crew with or without emergency physician, private transport, secondary transport), date of admission, stroke severity at presentation (measured by the NIHSS), age, pre-stroke modified Rankin Scale (mRS), sex, prior hypertension, prior diabetes mellitus, previous history of stroke, previous history of myocardial infarction (MI), hyperlipidemia, prior atrial fibrillation, other cardiac illness, prior peripheral artery disease, current smoker, history of alcohol abuse, time of presentation (admission during working hours or non-working hours). The variable selection procedure for the DNT model included the same variables as described for the ODT model but imaging modality (CT or MRI) and treating stroke center served as additional explanatory variables. Grouping of stroke centers was done according to rates of rt-PA treatment: thrombolysis rate <10%, 10–20%, >20%. We considered potential two-way interactions between all explanatory variables and relied on bottom-up model selection for determining significant interactions and explanatory variables. Due to the skewed distribution, the log-transformed ODT and DNT were used as target variables. The potential confounders age, stroke severity (measured by the NIHSS) and year of admission were coded using a higher degree polynomial to allow for continuous but non-linear, non-monotonous relationships. The appropriate degrees of the polynomials were determined with analyses of variance (ANOVA). To estimate the number of patients lost for rt-PA treatment due to PCS-related delay, we multiplied the number of PCS with the difference of the rates of rt-PA treatment: N_PCS_ × (R_ACS_ − R_PCS_) where *N* is the number of patients and *R* is the rt-PA-rate. rt-PA rates changed over the study period and we used data of the last two years only for this computation. All data were processed using the statistical environment *R*, version 2.15.2.

### Ethics approval

The study was approved by the Ethics Committee of the Medical University Vienna, Austria, Ref No. = EK-Nr.: 1485/2015.

## Results

### All patients

**Fig. 1:**
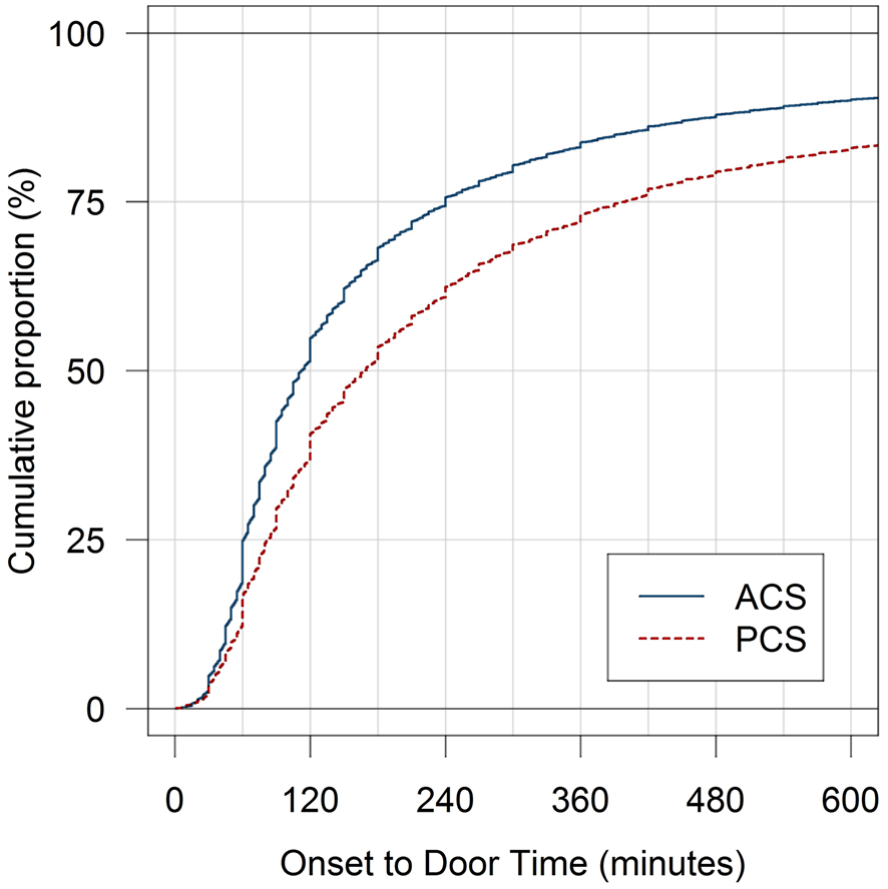
Association of ODT and infarct localization (PCS versus ACS)

Between March 2003 and February 2015, 71010 adult patients with IS were recorded in the ASUR, 11924 with PCS and 59086 with ACS. Patients with PCS had a significantly longer ODT compared to ACS: median: 170 min (25th percentile: 79 min, 75th percentile: 420 min) versus 110 (60, 240); *p* < 0.001 (Table 1). Demographics of all enrolled patients are shown in Table 1. In the multivariate analysis, patients with PCS lost on average 27 min compared to ACS before arrival to hospital (95% CI 23–31, *p* < 0.001; Supplementary Table I). Other variables significantly associated with a delayed ODT were stroke severity, type of transport, age, diabetes, atrial fibrillation and year of admission. Only 53.8% of patients with PCS arrived at hospital within 180 min as compared to 68.4% in patients with ACS (Fig. 1). The delays of ODT in patients with PCS were detectable over the entire study period (Supplementary Table I). Based on the proportion of rt-PA treatment in ACS between 2010 and 2014, we calculated that on average 100 patients with PCS per year potentially eligible for rt-PA treatment are lost due to prehospital delays (Supplementary Table III).

**Table 1:** Baseline characteristics of all patients stratified by infarct localization

	ACS (*n* = 59086)	PCS (*n* = 11924)
Age, years median (25th, 75th‰)	74.4 (64.6, 82.4)	70.6 (59.8, 79.1)
Male, sex (%)	30249 (51.2)	7119 (59.7)
NIHSS at baseline, median (25th, 75th‰)	4 (2, 10)	3 (1, 5)
Prior functional status mRS = 0, *n* (%)	39679 (67.2)	8814 (73.9)
Vascular risk factors, *n* (%)		
Hypertension	47494 (80.4)	9316 (78.1)
Diabetes mellitus	14944 (25.3)	2982 (25)
Hyperlipidemia	31898 (54)	6668 (55.9)
Atrial fibrillation	16543 (28)	2590 (21.7)
Smoking	1684 (17.1)	176 (17.2)
History of stroke	14137 (23.9)	2545 (21.3)
Previous myocardial infarction	5679 (9.6)	997 (8.4)
Previous peripheral artery disease	4091 (7)	808 (6.8)
Regular drinking	4638 (7.8)	914 (7.7)
Etiology		
Cardio-embolism, *n* (%)	15914 (26.9)	2551 (21.4)
Large artery disease, *n* (%)	7628 (12.9)	1295 (10.9)
Small artery disease, *n* (%)	15340 (26)	2746 (23)
Other, *n* (%)	1187 (2)	419 (3.5)
Undetermined, *n* (%)	19017 (32.2)	4913 (41.2)
Type of transport		
Ambulance without emergency physician	28067 (48.6)	5073 (42.8)
Ambulance with emergency physician	14350 (24.4)	2302 (19.4)
Private transport	8885 (15.1)	2517 (21.2)
Secondary transport^a^	7010 (11.9)	1966 (16.6)
Image modality		
Computer tomography	49098 (83.9)	8911 (75.3)
Magnet resonance imaging	5978 (10.2)	1782 (15.1)
Time of admission		
Working hours^b^	27932 (47.3)	5772 (48.4)
Non-working hours^c^	31152 (52.7)	6152 (51.6)

*NIHSS* National Institutes of Health Stroke Scale, *mRS* modified Rankin Scale, *ACS* anterior circulation stroke, *PCS* posterior circulation stroke

^a^Transport via another hospital to a stroke unit

^b^Monday–Friday 8–16 h

^c^Monday–Friday 16–8 h and weekend

### Patients treated with rt-PA

10854 (15.2%) patients with IS were treated with rt-PA (1022 with PCS and 9832 with ACS). Percentage of patients treated with rt-PA was lower in PCS as compared to ACS (8.6 versus 16.6%). Among rt-PA-treated patients, those with PCS were younger, more often male, had a lower NIHSS at baseline and atrial fibrillation was less often present (Table 2).

**Table 2:** Baseline characteristics of patients treated with rt-PA stratified by infarct localization

	ACS (*n* = 9832)	PCS (*n* = 1022)
Age, years median (25th, 75th‰)	73.8 (64, 81.4)	70.5 (59.1, 79.1)
Male, sex (%)	5073 (51.6)	629 (61.5)
NIHSS at baseline, median (25th, 75th‰)	9 (5, 16)	6 (4, 12)
Prior functional status mRS = 0, *n* (%)	7427 (75.5)	804 (78.7)
Vascular risk factors, *n* (%)		
Hypertension	7790 (79.2)	786 (76.9)
Diabetes mellitus	2050 (20.9)	215 (21)
Hyperlipidemia	5092 (51.8)	537 (52.5)
Atrial fibrillation	3150 (32)	230 (22.5)
Smoking	1684 (17.1)	176 (17.2)
History of stroke	1646 (16.7)	192 (18.8)
Previous myocardial infarction	871 (8.9)	95 (9.3)
Previous peripheral artery disease	503 (5.1)	48 (4.7)
Regular drinking	719 (7.3)	68 (6.7)
Etiology		
Cardio-embolism, *n* (%)	3451 (35.1)	265 (25.9)
Large artery disease, *n* (%)	1394 (14.2)	145 (14.2)
Small artery disease, *n* (%)	1695 (17.2)	194 (19)
Other, *n* (%)	163 (1.7)	32 (3.1)
Undetermined, *n* (%)	3129 (31.8)	386 (37.8)
Type of transport		
Ambulance without emergency physician	4256 (43.3)	436 (42.7)
Ambulance with emergency physician	4027 (41)	391 (38.3)
Private transport	484 (4.9)	74 (7.2)
Secondary transport^a^	1060 (10.8)	121 (11.8)
Image modality		
Computer tomography	8091 (82.7)	723 (70.9)
Magnet resonance imaging	1074 (11)	197 (19.3)
Time of admission		
Working hours^b^	4304 (43.8)	473 (46.3)
Non-working hours^c^	5528 (56.2)	549 (53.7)

*NIHSS* National Institutes of Health Stroke Scale, *mRS* modified Rankin Scale, *ACS* anterior circulation stroke, *PCS* posterior circulation stroke

^a^Transport via another hospital to a stroke unit

^b^Monday–Friday 8–16 h

^c^Monday–Friday 16–8 h and weekend

Without adjustment for confounding, OTT, ODT and DNT all were significantly longer among rt-PA-treated patients with PCS as compared to patients with ACS: OTT: median: 145 min (25th percentile: 108 min, 75th percentile: 195 min) versus 120 (95, 165), *p* < 0.001; ODT: median 80 (55, 120) versus 72 (50, 110), *p* < 0.001; DNT: median 57 (35, 90) versus 45 (30, 67), *p* < 0.001 (Table 2).

**Fig. 2:**
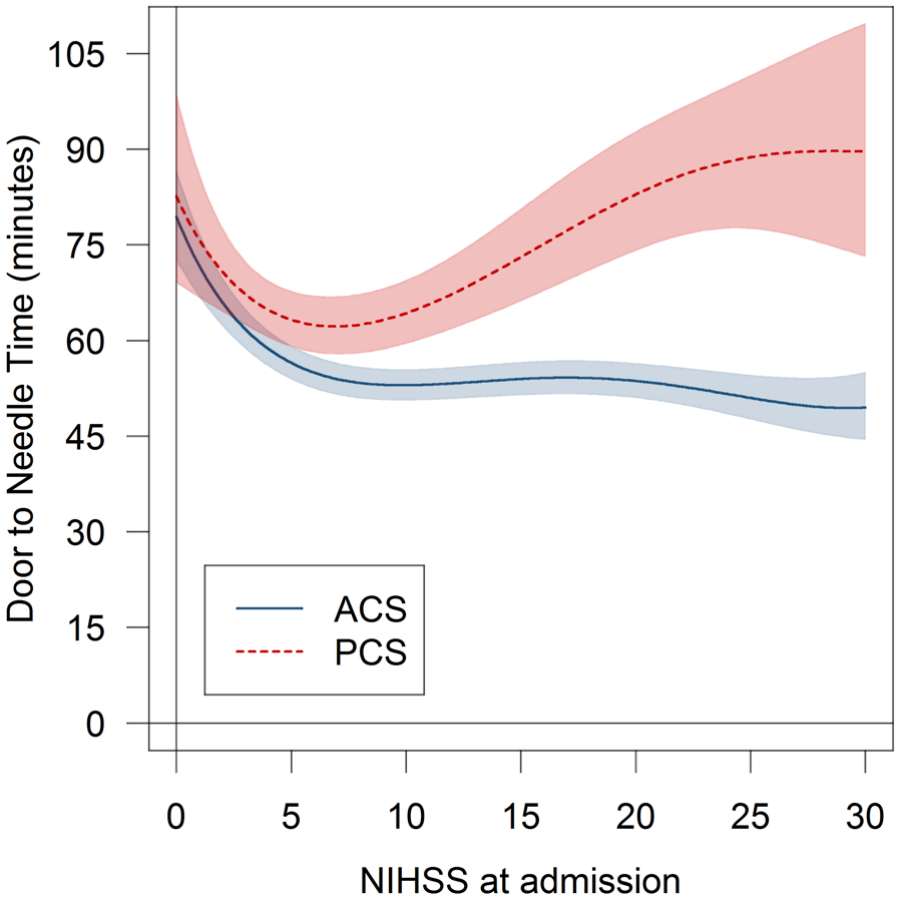
Associations of localization [posterior circulation stroke (PCS) versus anterior circulation stroke (ACS)], stroke severity (NIHSS) and the door-to-needle time (DNT). *Graphs* are based on the adjusted model displayed in Supplementary Table II-R

PCS was significantly associated with a delay in the DNT after adjustment for confounding factors. The DNT was on average 13 min longer in patients with PCS (95% CI 10–17, *p* < 0.001, Supplemental Table II-R). Other variables significantly associated with a prolonged DNT were stroke severity, treating stroke center, type of imaging, type of transport and year of admission. In addition, we detected a significant interaction of the NIHSS and PCS (Supplemental Table II-R). In an adjusted analysis of associations of DNT, stroke severity and localization, we detected a positive correlation between stroke severity and treatment delay except for patients with mild stroke (Fig. 2). The delays of DNT in patients with PCS were detectable over the entire study period (Suppl. Table II-R).

In multivariable analysis of patients treated with rt-PA, the association between ODT and PCS lost significance (data not shown).

## Discussion

In this analysis of nationwide data of the ASUR, we detected delays in pre- and intra-hospital time intervals in patients with acute ischemic stroke within the posterior circulation. As symptoms of PCS can mimic other disorders, they can be misinterpreted easily not only by non-neurologists but also by neurologists [11].

### ODT

Screening tools such as the Face Arm Speech Test (FAST) developed for prehospital identification of stroke patients are less sensitive for identification of PCS compared to ACS [21]. This lack of early identification of symptoms might account for delays in prehospital management leading ultimately to ineligibility for thrombolytic treatment. However, there is limited evidence from the literature whether stroke localization is related to delay in preclinical time intervals so far: two prior studies both from the pre-rt-PA era [22, 23] did not detect significant differences and only one of them stratified stroke type according to the OCSP classification [23]. In our study, we detected in all patients, irrespective of rt-PA treatment, substantially longer durations of prehospital patient management in patients with PCS as compared to patients with ACS. On average, patients with PCS lost 27 min. Relevantly, only half of patients with PCS arrived within 3 h in hospital compared to 2/3 in ACS. Based on these data, we estimated that on average 100 patients with PCS per year potentially eligible for rt-PA treatment might have been missed due to prehospital delays. As a consequence, faster prehospital management could result in an almost twofold increase of the thrombolysis rate in patients with PCS.

In addition, prehospital loss of patients potentially eligible for rt-PA treatment might also explain the observed lack of association of infarct localization (i.e., ACS versus PCS) and the ODT in the selected group of patients treated with rt-PA, which is in agreement with previous work [12].

The delay of patients with PCS reflects that diagnostic tools used in the prehospital setting do not focus on PCS-related symptoms. Therefore, both education and optimized screening tools need to take typical symptoms of PCS into account. For instance, adding elements used by the three-step bedside examination for differential diagnosis of acute vestibular syndrome may increase detection rates of PCS [24]. Moreover, addition of ataxia testing as well as visual field testing may substantially increase specificity and sensitivity of the FAST-test [21]. Furthermore, most symptoms preceding PCS—so-called transient neurological attacks (TNAs)—do not satisfy traditional definitions of TIAs [16]. Therefore, evaluation of TNAs could be useful in screening for PCS. Randomized studies testing prehospital screening tools that include those tests for PCS are warranted.

### DNT

In patients with PCS, the DNT was on average 13 min longer compared to patients with ACS. This is a substantial delay, given that even small reductions in time-to-thrombolysis translate into a significant gain of healthy lifetime [14]. While we did not observe any differences in DNT in patients with mild stroke, we detected a significant delay in DNT for patients with PCS with moderate and severe deficits. In those with the most severe strokes, this observation might be explained by a greater necessity for intensive care measures in patients with PCS. However, the observed delay in DNT in PCS did not only include patients with the most severe strokes but also those with moderate severity. There are some possible explanations for this finding: First, the NIHSS has limitations for assessment of PCS and symptoms not measured by the NIHSS might contribute to time delays [3, 5, 12]. This is reflected by our observation of overall lower NIHSS scores in patients with PCS, which is in agreement with previous work [3, 5, 12, 25, 26]. It is, therefore, possible that clinical deficits have been underestimated by the NIHSS [25, 26]. Second, incorrect patient triage might have contributed to the delay in DNT. Even if in the ASUR, the majority of patients subsequently receiving treatment with rt-PA arrived via ambulance making triage errors in the ER less likely, we cannot exclude that triage errors or errors in hospital pre-notification may have contributed to delays in DNT [28]. Third, in PCS, MRI has been more often performed which might have contributed to the observed delay; however, the prolonged DNT was irrespective of type of imaging in the multivariate analysis.

Time to treatment has been associated with clinical outcome after ischemic stroke in clinical studies and large clinical registers [15, 29, 30]. Reductions in prehospital time intervals have been associated with better functional outcome and a shorter DNT has been associated with lower in-hospital mortality as well as lower rates of symptomatic intracranial hemorrhage [27, 31]. Therefore, we believe that the pre- and intra-hospital delays we observed in patients with PCS are clinically relevant. Our study outnumbers previous studies analyzing time intervals stratified by infarct localization using data of a nationwide cohort of patients (Supplementary Table IV). In addition, previous studies focused exclusively on rt-PA-treated patients and did not analyze ODT in untreated patients; therefore, estimates of patients potentially lost for rt-PA-treatment were not possible.

In contrast to previous work suggesting greater ischemic tolerance in posterior circulation as compared to anterior cerebral circulation [32], more recent data from the same group could not support this hypothesis [33]. In a post hoc analysis of the IST-3 trial, no differences were found between ACS and PCS; however, this subgroup analysis was certainly underpowered [34]. Therefore, at the current stage, there is no evidence that the time-window for intravenous thrombolysis is different in PCS and ACS and delays might have similar negative effects on outcome and should be avoided irrespective of stroke localization.

The observed in pre- and intra-hospital delays of our study may have implications for daily routine and demonstrate the need for improvement in organization of stroke pathways. Pre- and intra-hospital screening tool and triage systems need to implement typical symptoms of PCS to alert stroke code protocols in a timely manner.

Our study has limitations. First, data collected in registries do not compensate for randomized studies, as occurrence of selection bias cannot be excluded. However, entry of patient data into the ASUR is mandatory and the registry is part of a governmental quality assessment program. Hence, we assume that almost all patients undergoing treatment with rt-PA are included into the ASUR. Second, diagnosis of PCS was clinically determined according to the OCSP classification [19]. Importantly, the OCSP classification has an excellent sensitivity and specificity for patients with strokes in the posterior circulation; correct classification rates of more than 85% have been previously reported [35]. Nevertheless, we cannot exclude that some patients with lacunar strokes in the brainstem might have been classified as lacunar syndrome according to the OCSP classification [19, 35]. Third, we were missing data on affected vessel pathology, since CTA or MRA are not mandatory for diagnosis in the ASUR. Therefore, it was not possible to focus on specific conditions, such as basilar artery occlusion. However, basilar artery occlusion was the focus of large, multinational registries [36–39] and treatment options in basilar occlusion are currently evaluated in the ongoing BASICS study [40]. Fourth, as only patients admitted to stroke units were included into the ASUR, we were not able to analyze patients misdiagnosed prior to admission. However, as in all centers participating in the ASUR recruitment is done exclusively by neurologists specialized in acute stroke care, we assume that false-negative rates were low. Fifth, we cannot exclude that some stroke patients have not been admitted to stroke units but directly to general neurological wards. This might have affected predominantly patients with minor symptoms or those with very long ODTs.

## Conclusion

In this large nationwide cohort, time until arrival to hospital was longer and treatment with rt-PA was delayed in patients with PCS as compared to patients with ACS. Whether the observed time treatment delays have an impact on the outcome of PCS needs to be determined in future studies. Furthermore, prospective studies, particularly in the prehospital setting, are needed to determine factors leading to delays specifically in patients with PCS. Scales focusing on clinical symptoms related to the posterior circulation might enable more efficient patient triage in the future.

## Electronic supplementary material

Below is the link to the electronic supplementary material.

Supplementary material 1 (PDF 198 kb)
