# Supplementary material for: Prehospital and intra-hospital time delays in posterior circulation stroke: results from the Austrian Stroke Unit Registry
Source: J Neurol. 2016 Nov 7;264(1):131–8. doi: 10.1007/s00415-016-8330-x (PMC5225195; doi:10.1007/s00415-016-8330-x)
Supplement: Supplementary file 1 — Supplementary material 1 (PDF 198 kb) [file 415_2016_8330_MOESM1_ESM.pdf]

# Online-Only Supplemental Data

## Prehospital and intrahospital time delays in posterior circulation stroke: results from the Austrian Stroke Unit Registry

[a]Peter Sommer, MD, [b]Leonhard Seyfang, MoS, [b]Alexandra Posekany, PhD, [c]Julia Ferrari, MD, [c]Wilfried Lang, MD, [a]Elisabeth Fertl, MD, [d]Wolfgang Serles, MD, [e]Thomas Töll, MD, [e]Stefan Kiechl, MD, [d]Stefan Greisenegger, MD.

*[a]Department of Neurology, Krankenanstalt Rudolfstiftung Vienna, Austria*

*[b]Danube University Krems & Gesundheit Österreich GmbH/BIQG, Vienna, Austria*

*[c]Department of Neurology, Krankenhaus Barmherzige Brüder, Vienna Austria*

*[d]Department of Neurology, Medical University of Vienna, Austria*

*[e]Department of Neurology, Medical University of Innsbruck, Austria*

**SUPPLEMENTAL TABLES**

Supplemental Table I

Supplemental Table II-R

Supplemental Table III

Supplemental Table IV

**SUPPLEMENTAL TABLE I:** Linear regression model[a] of the log-transformed ODT (n=43778).

| Variable | Coef. | SE | p | Minutes[b] |
|---|---|---|---|---|
| Intercept | 4.866 | 0.014 | | 130 (126 to 133) |
| Posterior circulation stroke | 0.191 | 0.013 | <0.001 | 27 (23 to 31) |
| Age polynomial 1[c] | 2.050 | 0.974 | 0.035 | |
| Age polynomial 2 | -6.379 | 0.910 | <0.001 | |
| Type of transport | | | | |
| Ambulance with emergency physician | -0.247 | 0.011 | <0.001 | -28 (-31 to -26) |
| Private transport | 0.408 | 0.015 | <0.001 | 65 (60 to 71) |
| Secondary transport[d] | 0.406 | 0.015 | <0.001 | 65 (60 to 71) |
| NIHSS polynomial 1[c] | -25.653 | 0.996 | <0.001 | |
| NIHSS polynomial 2 | 2.282 | 0.910 | 0.012 | |
| NIHSS polynomial 3 | 10.024 | 0.903 | <0.001 | |
| NIHSS polynomial 4 | -11.041 | 0.901 | <0.001 | |
| NIHSS polynomial 5 | 8.131 | 0.899 | <0.001 | |
| NIHSS polynomial 6 | -3.841 | 0.899 | <0.001 | |
| Diabetes mellitus | 0.091 | 0.011 | <0.001 | 12 (9 to 15) |
| Atrial fibrillation | -0.089 | 0.011 | <0.001 | -11 (-14 to -8) |
| Year of admission polynomial | -0.008 | 0.002 | <0.001 | -1 (-1 to -1) |

Coef: coefficient of model, SE: standard error of coefficients, NIHSS: national institute of health stroke scale.

[a]The overall significance of the multivariate model was tested by ANOVA, p value<0.001, coefficient of determination $R^2$: 0.12. Reference levels for the model were: stroke localization: *ACS*, type of transport: *transport without emergency physician*, diabetes: *No presence of diabetes*, atrial fibrillation: *No presence of atrial* fibrillation, year of admission: *2003.*

[b]Minutes: retransformed coefficients calculated as: $e^{I+C} – e^{I}$ where I is the coefficient of the Intercept, C is the current coefficient and e is Euler's number.

[c]The variables age and NIHSS were included into the model in form of polynomials and no stratification or grouping has been applied to the variables. The digits 1-6 of the variable name represent the weights of the corresponding terms of the polynomial.

[d]Transport to stroke unit via another hospital

**SUPPLEMENTAL TABLE II-R:** Multivariate linear regression model of the log-transformed DNT (n=10626).

| Variable | Coefficient | SE | p | Minutes[a] |
|---|---|---|---|---|
| Intercept | 4.002 | 0.021 | | 55 (53 to 57) |
| Posterior circulation stroke | 0.218 | 0.024 | <0.001 | 13 (10 to 17) |
| Treating stroke center[b] | | | | |
| Thrombolysis rate 10-20% | -0.133 | 0.021 | <0.001 | -7 (-9 to -5) |
| Thrombolysis rate >20% | -0.249 | 0.022 | <0.001 | -12 (-14 to -10) |
| Type of transport | | | | |
| Ambulance with emergency physician | -0.082 | 0.014 | <0.001 | -4 (-6 to -3) |
| Private transport | 0.224 | 0.031 | <0.001 | 14 (10 to 18) |
| Secondary transport[c] | -0.149 | 0.023 | <0.001 | -8 (-10 to -5) |
| Magnetic resonance imaging | 0.374 | 0.020 | <0.001 | 25 (22 to 28) |
| NIHSS polynomial 1[d] | -4.162 | 0.701 | | |
| NIHSS polynomial 2 | 4.202 | 0.821 | | |
| NIHSS polynomial 3 | -1.707 | 0.884 | | |
| NIHSS polynomial 4 | 3.839 | 0.813 | | |
| Year of admission polynomial 1[d] | -5.058 | 0.627 | | |
| Year of admission polynomial 2 | 1.261 | 0.616 | | |
| PCS x NIHSS polynomial 1 | 12.679 | 2.186 | | |
| PCS x NIHSS polynomial 2 | -0.714 | 1.794 | | |
| PCS x NIHSS polynomial 3 | -2.997 | 1.737 | | |
| PCS x NIHSS polynomial 4 | -1.293 | 1.657 | | |

SE: standard error of coefficients; NIHSS: national institute of health stroke scale, MRI: magnetic resonance imaging, mRS: modified Rankin Scale

The overall significance of the multivariate model was tested by ANOVA, p value<0.001, coefficient of determination $R^2$=0.146; Reference levels for the model were: stroke localization: *ACS*, treating stroke center: *Thrombolysis rate <10%*, type of transport: *transport without emergency physician*, image modality: *CT*, year of admission: *2003*

[a]Minutes: retransformed coefficients calculated as: $e^{I+C} – e^{I}$ where I is the coefficient of the Intercept, C is the current coefficient and e is Euler's number.
[b]Grouping of stroke centers was done according to the experience (rate of rt-PA-treatment): thrombolysis rate <10%, 10-20%, >20%.
[c]Transport via another hospital to a stroke unit.
[d]NIHSS and year of admission were included into the model as polynomials and no stratification or grouping has been applied to the variables. The digits of the variable name represent the weight of the corresponding terms of the polynomial. Significance of the polynomial variables NIHSS and year of admission were tested by ANOVA, p value for both<0.001.

**SUPPLEMENTAL TABLE III:** Potential lost patients with PCS eligible for thrombolysis between 2010 and 2014.

| Year | PCS[a] | rt-PA rate[b] | Patients potentially lost[c] |
|---|---|---|---|
| 2010 | 1232 | 11.6% | 81 |
| 2011 | 1322 | 10.7% | 118 |
| 2012 | 1213 | 12.2% | 85 |
| 2013 | 1367 | 9.9% | 125 |
| 2014 | 1323 | 12.9% | 89 |

[a]Patients with posterior circulation stroke.

[b]Rate of patients with PCS treated with rt-PA.

[c]Patients potentially lost given the ODT-distribution of patients with anterior circulation stroke.

**SUPPLEMENTAL TABLE IV:** Comparison of previous studies analyzing time-intervals in stroke patients stratified by localization.

| | Number of analyzed patients | | Number of centers |
|---|---|---|---|
| | ACS | PCS | |
| Förster et al.[3] | 198 | 30 | 1 |
| Sarikaya et al.[5] | 798 | 95 | 3 |
| Sarraj et al.[11] | 221 | 31 | 1 |
| This study (All) | 59086 | 11924 | 36 |
| This study (rtPA-treated patients) | 9832 | 1022 | 36 |
